# The Mean Platelet Volume in Patients with Retinal Vein Occlusion

**DOI:** 10.1155/2013/236371

**Published:** 2013-05-28

**Authors:** Alparslan Şahin, Muhammed Şahin, Harun Yüksel, Fatih Mehmet Türkcü, Yasin Çınar, Abdullah Kürşat Cingü, Şeyhmus Arı, İhsan Çaça

**Affiliations:** Department of Ophthalmology, School of Medicine, Dicle University, 21280 Diyarbakır, Turkey

## Abstract

*Background*. The aim of this study was to investigate the mean platelet volume (MPV) of patients with retinal vein occlusion (RVO). *Methods*. Hundred and ninty-three patients with the diagnosis of RVO and 83 healthy control subjects were included in this retrospective study. Retinal vein occlusion was diagnosed based on clinical examination. All patients and control subjects underwent complete ocular examination. MPV, hematocrit, hemoglobin, and platelet count of the participants were recorded. The data of patients with RVO was compared with the control subjects. *Results*. Patients with RVO had significantly higher MPV values (8.19 ± 1.22 fL) compared with the control subjects (7.68 ± 1.11 fL) (*P* = 0.004). No significant difference was found in platelet counts between RVO group and the control group (275.77 ± 70.87 10^9^/L and 261.96 ± 59.40 10^9^/L, resp., *P* = 0.161), Mean platelet volume was an independent predictor of RVO (odds ratio (OR) = 1.43; 95% confidence interval (CI) = 1.09–1.89; *P* = 0.011). *Conclusion*. Our results demonstrated that the MPV values were significantly higher in patients with RVO, suggesting that larger platelets may contribute to the pathogenesis of the RVOs.

## 1. Introduction

Retinal vein occlusion (RVO) is a major sight-threatening disease of the retina that causes vision loss, blindness, and neovascular glaucoma, especially in elderly patients. According to the location of the obstruction, the RVO can be classified as central retinal vein occlusion (CRVO) and branch retinal vein occlusion (BRVO). The prevalence of CRVO and BRVO is 0.08% and 0.44%, respectively [1]. 

The pathogenesis of the RVO is not clear. However, systemic conditions such as hypertension, diabetes mellitus, hypercholesterolemia, pregnancy, use of oral contraceptive agents, and inherited thrombophilia have been shown to be related with RVO [2]. Increased intraocular pressure and/or glaucoma have established local risk factors for RVO [3]. Management of vision loss and neovascularization is still challenging. Both BRVO and CRVO variably cause vision loss depending on the retinal ischemia and macular edema [4]. 

Platelets have an important role in the pathogenesis of thrombo-occlusive disease. Mean platelet volume (MPV) is an indicator of platelet size and has been known to be a marker of platelet activity. Large platelets are more reactive than small platelets and produce more thromboxane A_2_, express more glycoprotein Ib and glycoprotein IIb/IIIa receptors, and aggregate more easily [5, 6].

Increased values of MPV have been shown as a risk factor for stroke and myocardial infarction [7, 8]. In a recent study, significant correlation was found between the degree of retinopathy and the mean values of MPV in diabetic patients [9]. To the best of our knowledge, we did not find any article in the Medical Literature in English that studied the MPV in patients with RVO. The purpose of the current study was to investigate the MPV levels and platelet counts in patients with RVO. 

## 2. Materials and Methods 

All subjects underwent a full ocular examination using slit lamp biomicroscopy and fundoscope. Patients who were diagnosed as any type of RVO between January 2006 and December 2011 were enrolled. CRVO was diagnosed on the basis of the presence of retinal hemorrhages combined with retinal vein dilatation in four retinal quadrants. Patients with venous dilation and tortuosity with flame-shaped and dot-blot hemorrhages in a wedge-shaped region were diagnosed as BRVO.

The patients and the subjects with any systemic disease other than hypertension [10] were excluded from the study. Patients with diabetes mellitus, anemia (hematocrit below 38%) [11], any cardiovascular disease, such as congestive heart failure, and heart valve disease treated with anticoagulant, stroke, chronic renal failure, history of alcohol consumption, and history of smoking were excluded from the study. Patients with glaucoma and with a history of any ocular surgery or trauma were also excluded. 

Control group consisted of healthy subjects who had undergone routine ophthalmic examination. Best corrected visual acuities and intraocular pressures of all subjects were recorded. Fundus fluorescein angiography was performed in patients with RVO.

Blood samples were taken at the time of diagnosis of RVO. Complete blood count samples which were drawn into vacutainer tubes containing 0.04 mL of the 7.5% K3 salt of EDTA were analyzed within an hour after sampling with a commercially available analyzer (CELL-DYN 3700, Abbott Diagnostics, Abbott Park, IL, USA). MPV, platelet count, hemoglobin, and hematocrit parameters of the subjects were recorded.

The study protocol was approved by the Ethic Committee of Dicle University School of Medicine, and the study was conducted in accordance with the Declaration of Helsinki. 

### 2.1. Statistical Analyses

All values were given as mean ± SD. Statistical analyses were performed using SPSS versus 11.5 statistical package program. The *Kolmogorov-Smirnov* test was applied to test the distribution pattern of each data. The *Student's t-test* was used for normally distributed data in group comparisons. A *P* value less than 0.05 was considered significant. *Receiver-operating characteristic (ROC)* analyses were used to determine the cutoff values and the sensitivity/specificity of the MPV. *Univariate logistic regression* analysis was used to assess the associations among MPV, hematocrit and hemoglobin levels, hypertension, age, and gender with RVO.

## 3. Results

Patients with any type of RVO were consecutively recruited at the Dicle University, School of Medicine, Department of Ophthalmology. Totally, 193 patients with RVO were eligible for the study. Control group consisted of 83 subjects. The mean age of the RVO group and the control group was 60.8 ± 12.4 and 61.6 ± 15.8 years, respectively. Male-to-female ratio was 98/95 in the RVO group, and 38/45 in the control group. There were no statistical differences in age and sex between groups (*P* > 0.05) (Table 1). There was no significant difference in the control (36 of 83) and RVO (89 of 193) groups, according to presence of hypertension. There was no difference between groups regarding antihypertensive drug use.

The mean MPV was significantly higher in patients with RVO than the control group (8.19 ± 1.22 fL versus 7.68 ± 1.11 fL, *P* = 0.004). The mean platelet count was slightly higher in the control group compared to RVO group, 275.77 ± 70.87 10^9^/L and 261.96 ± 59.40 10^9^/L, respectively (*P* = 0.161). Logistic regression analysis showed that MPV was also an independent predictor of RVO (odds ratio (OR) = 1.43; 95% confidence interval (CI) = 1.09–1.89; *P* = 0.011).

**Figure 1:**
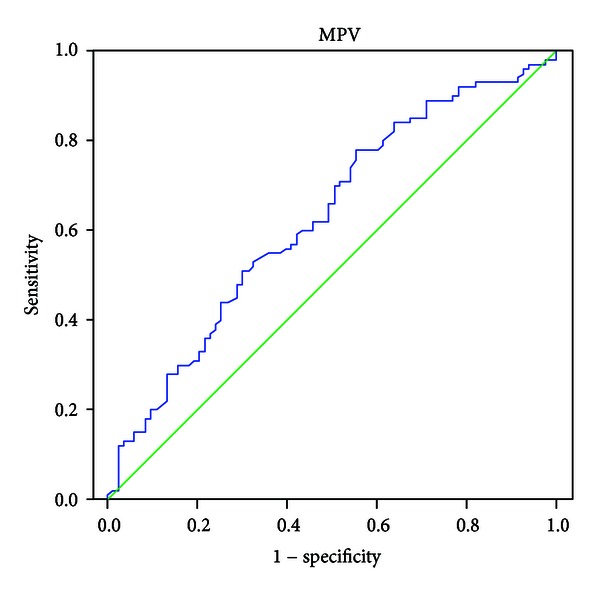
Receiver-operating characteristic curves for predictors of retinal vein occlusion. The MPV values area under the curve, 0.628; green line.

In the ROC analysis performed, when the control group and patients with RVO were compared, the cutoff value was 8.00 (area under curve (AUC): 0.628), sensitivity was 56.1%, and specificity was 60.2% for the patients with RVO (*P* = 0.003) (Figure 1).

## 4. Discussion

The current study showed the higher levels of MPV in patients with RVO. To the best of our knowledge, this is the first paper, shows that relationship between higher MPV values in patients with RVO.

Retinal vein occlusions are the most common cause of vision loss, after diabetic retinopathy, in the elderly patients [12]. Vision is decreased depending on the amount of retinal ischemia and macular edema. The pathogenesis of RVO is multifactorial and has not yet been elucidated. Several risk factors such as age, smoking, hypertension, diabetes mellitus, hyperlipidemia, and glaucoma were attributed in the etiology of RVO [12]. 

The platelets have an important role in the pathogenesis of vascular diseases. MPV is the indicator of the size and activity of platelets. Increased values of MPV have been shown as a risk factor for stroke [7]. Relationship between increased MPV with deep venous thrombosis [13], acute myocardial infarction [14], and acute ischemic cerebrovascular events [15] was also reported. Since larger platelets store and release larger amounts of serotonin and *β*-thromboglobulin and produce more thromboxane A_2_, they are more reactive and prone to aggregation [5, 6]. 

The roles of platelets in RVOs were also studied. Leoncini et al. reported increased platelet response to thrombin in patients with RVO [16]. They suggested that platelet hyperaggregability inducing thrombus formation might be an important factor in the onset and/or development of RVO. Increased platelet activation induced by collagen [17], increased levels of platelet factors [18], and platelet hyperaggregability [19] were reported in RVOs.

As larger platelets are haemostatically more active, they produce more prothrombotic factors [20]. Bath and Butterworth reported that the platelet hyperactivity results in an increase in MPV [21]. Larger platelets aggregate easier than smaller ones [6]. In our study, the MPV values were significantly higher compared to control group which may contribute to the pathogenesis of RVO. Logistic regression analysis revealed that MPV is an independent predictor for RVO. Presence of high MPV in these patients may increase the risk of RVO.

MPV was studied in a few ocular vascular disorders. Ateş et al. reported a significant increase in MPV in patients with diabetic retinopathy [9]. They found a correlation between the severity of diabetic retinopathy and MPV values. Ricart et al. found that MPV was not related with posterior uveitis in Behçet's disease [22]. 

Several studies also demonstrated that MPV is elevated in venous occlusive diseases such as deep venous thrombosis [13, 23], sinus thrombosis [24], and pulmonary thromboembolism [25]. A higher level of MPV is seemed to be a risk factor for vascular occlusive diseases.

The limitations of our study are lack of systolic and diastolic pressure of the subjects, lack of body mass index and lipid profile of the patients, and the retrospective nature of the study. In the current study, the results demonstrate only the hematologic status at the acute stage of RVO within a week. Therefore, these results may not reflect the status of these patients over long periods.

## 5. Conclusion

MPV was significantly higher in patients with RVO. Despite the retrospective nature of our study, we suggest that MPV may be used as a predictive tool for identifying risk for RVOs in the future. Further studies are needed to confirm the predictive value of MPV for the RVO risk.

## Figures and Tables

**Table 1:** The demographic and clinical features of the RVO and control groups.

	Retinal vein occlusions	Control	*P* value
Age (years)	60.8 ± 12.4	61.6 ± 15.8	0.65
Gender (M/F)	98/95	38/45	0.27
Hypertension	89/193	36/83	0.11
Hb (g/dL)	13.8 ± 1.6	13.6 ± 1.4	0.41
Hct (%)	40.7 ± 4.5	40.1 ± 4.0	0.43
Plt (K/uL)	262.0 ± 59.4	275.8 ± 70.9	0.16
MPV (fL)	8.19 ± 1.22	7.68 ± 1.11	**0.004**
IOP (mmHg)	13.88 ± 4.03	13.22 ± 3.02	0.19
Visual acuity (snellen)	0.41 ± 0.30	1.00 ± 0.0	**<0.001**

Hb: hemoglobin, Hct: hematocrit, Plt: platelet count, MPV: mean platelet volume, IOP: intraocular pressure.
